# Hidden causal inference delineates dynamic lncRNA regulation in autism spectrum disorder

**DOI:** 10.1093/bib/bbag541

**Published:** 2026-09-30

**Authors:** Junpeng Zhang, Xuemei Wei, Chunwen Zhao, Zidan Hu, Shujie Fan

**Affiliations:** School of Engineering, Dali University, No. 2 Hongsheng Road, Dali, Yunnan 671003, China; School of Engineering, Dali University, No. 2 Hongsheng Road, Dali, Yunnan 671003, China; School of Engineering, Dali University, No. 2 Hongsheng Road, Dali, Yunnan 671003, China; School of Engineering, Dali University, No. 2 Hongsheng Road, Dali, Yunnan 671003, China; School of Engineering, Dali University, No. 2 Hongsheng Road, Dali, Yunnan 671003, China

**Keywords:** lncRNA, mRNA, single-nucleus RNA sequencing, causal inference, dynamic lncRNA regulation, autism spectrum disorder

## Abstract

Long non-coding RNAs (lncRNAs) are increasingly implicated in autism spectrum disorder (ASD), yet their dynamic regulatory roles remain poorly characterized. Conventional static causal inference methods are limited in capturing the dynamic interplay of lncRNAs across diverse brain biological contexts. Here, we develop DCRNet (Dynamic Causal Regulation Network), a hidden causality-based framework to infer lncRNA regulatory relationships in ASD. Our analysis reveals that dark causality, representing a hybrid form of positive and negative interdependencies, is prevalent in lncRNA-mediated regulation. Moreover, these dynamic regulatory patterns emerge across multiple biological contexts, and eight ASD susceptibility lncRNAs may be linked to disrupted gene regulatory programs. We also indicate that lncRNA expression signatures distinguish sex and cell type with high accuracy, offering candidate diagnostic biomarkers. Notably, 20 immune-related lncRNAs are predicted to be involved in neuroimmune regulation. Additionally, cell–cell interaction networks show notable differences between ASD and control cohorts. Furthermore, DCRNet performs comparably to the other two causal inference methods and outperforms a correlation-based approach in capturing dynamic lncRNA regulation. This study provides a framework for delineating dynamic lncRNA regulation and suggests their potential relevance to ASD pathophysiology.

## Introduction

Autism spectrum disorder (ASD) is a group of neurodevelopmental conditions characterized by two core deficits: impairments in verbal and nonverbal communication and restricted/repetitive patterns of behavior, interests, and activities [1]. Although the etiology of ASD remains unclear, current consensus implicates that it is caused by a combination of genetic and environmental factors. These genetic and environmental triggers induce cellular dysfunction, leading to neuroinflammation [2], excitation/inhibition imbalance [3], and white matter abnormalities [4]. Traditional ASD diagnosis relies on clinical symptoms, including social communication, social interactions, and behavioral/sensory processing. However, these clinical symptoms demonstrate limited applications across sex, age, and intellectual ability. Furthermore, core clinical symptoms (e.g. impaired social communication, restricted behaviors) are emerged clearly only after age three, delaying early intervention opportunities. Emerging diagnostic methods leverage ASD susceptibility genes as biomarkers for ASD prediction. These susceptibility genes frequently harbor mutations driven by sex-linked modifiers, epigenetic regulation, copy number variations, environmental exposures, or double-hit mutational events [5]. Biomarker-based molecular diagnosis is unaffected by age, sex, or intellectual ability, facilitating earlier detection and intervention [5–7]. This paradigm shift toward molecular diagnosis holds promise for improving ASD intervention outcomes.

Generally, ASD biomarkers are predominantly limited to protein-coding genes, as exemplified by the SFARI [8], AutismKB [9], and AutDB [10] databases. Recent studies, however, reveal critical roles for non-protein-coding genes in ASD pathogenesis [11]. For example, the RNADisease database [12] systematically documents non-coding RNAs (ncRNAs) related to ASD. These ncRNAs modulate gene expression through DNA-level, pre-transcriptional, transcriptional, post-transcriptional, translational, and post-translational processes, thereby contributing to ASD progression [13]. Among the diverse classes of ncRNAs, long non-coding RNAs (lncRNAs), defined as RNA transcripts exceeding 200 nucleotides, have emerged as important regulators in ASD [11]. Emerging evidence suggests that several lncRNAs (e.g. *H19, NEAT1, MALAT1, MEG3, PVT1, MIR600HG, CSNK1A1P1, PCAT-1, PCAT-29, lincRNA-ROR, LINC-PINT, lincRNA-p21*) serve as promising non-invasive biomarkers by modulating synaptic plasticity and neurodevelopmental pathways [14–21]. Investigations into lncRNA-mediated regulation offer novel insights into the molecular pathogenesis underlying ASD and contribute to discovering novel lncRNAs as therapeutic targets for ASD [22]. Nevertheless, the functional characterization of lncRNAs in gene regulation and their clinical potential as ASD biomarkers remains underexplored.

Since lncRNAs are characterized by dynamic regulation, low expression, and low conservation, they exhibit a great diversity in gene regulatory mechanisms [23, 24]. In ASD, the mechanistic complexity of gene regulation suggests that the lncRNA regulation is inclined to be dynamic across various conditions. However, current approaches generally fail to capture conditional shifts in lncRNA regulation, limiting insights into how condition-specific perturbations drive ASD pathogenesis. While Cycle [25] enables dynamic modeling of lncRNA regulatory networks across diverse cell types in ASD, it remains restricted to inferring cell-type-resolved correlative regulation, neglecting other ASD biological contexts (e.g. diagnosis, region, age, and sex) and causal regulation by lncRNA. However, such as changes in diagnosis, region, age, and sex, most ASD mechanisms can be directly affected, and the dependency of lncRNA causal mechanisms on such biological contexts should be learned.

To address this gap, we develop a hidden causality-based framework named DCRNet (Dynamic Causal Regulation Network) to delineate dynamic causal regulation by lncRNAs across diverse ASD biological contexts, such as diagnosis, region, age, sex, and cell type. For a comprehensive understanding of lncRNA causal regulation in ASD, DCRNet infers three types of causal relationships mediated by lncRNAs: positive, negative, and dark causality (a hybrid form of positive and negative interdependencies). This mixed form can arise when an lncRNA dynamically switches its regulatory direction on the same target gene over developmental time. For instance, the *METEOR* lncRNA locus promotes *EOMES* expression in pluripotent stem cells but represses *EOMES* through its embedded DNA element upon neuronal differentiation [26]. Similarly, the *HAUNT* lncRNA transcript prevents premature *HOXA* activation in embryonic stem cells, while during differentiation its DNA locus switches to facilitate *HOXA* gene activation [27].

Our analysis reveals that “dark causality” is prevalent in lncRNA-mediated regulation, identifies eight ASD susceptibility lncRNAs associated with disrupted gene regulatory programs, and suggests that potential lncRNA biomarkers could classify sex and cell type. Furthermore, we identify 20 immune-related lncRNAs that may participate in neuroimmune regulation and observe notable differences in cell–cell interaction networks between ASD and control cohorts. Collectively, DCRNet provides a useful framework for stratifying ASD etiologies and offers insights that may inform therapeutic strategies targeting dynamic causal regulatory states.

## Materials and methods

### Single-nucleus RNA sequencing data in autism

We obtained single-nucleus RNA sequencing (snRNA-seq) data from cortical tissues, including the anterior cingulate cortex (ACC) and prefrontal cortex (PFC), of patients with autism, accessed from the Sequence Read Archive (SRA) under accession number PRJNA434002 [28]. We utilized gene annotation information from HGNC (HUGO Gene Nomenclature Committee) [29] to further divide RNA transcripts into lncRNAs and mRNAs. Given that low-expression genes might lack biological significance, we restricted our analysis to genes demonstrating expression levels above the mean expression value across all cells. In total, we have identified 813 lncRNAs and 5133 mRNAs exhibiting high expression levels across 104 559 cells (including 52 003 autism cells and 52 556 control cells). Based on the known expression of cell type markers [28], the 52 003 autism cells and 52 556 control cells are annotated into 17 cell types, including fibrous astrocytes (AST-FB), protoplasmic astrocytes (AST-PP), oligodendrocyte precursor cells (OPC), layer 2/3 excitatory neurons (L2/3), layer 4 excitatory neurons (L4), layer 5/6 corticofugal projection neurons (L5/6), layer 5/6 cortico-cortical projection neurons (L5/6-CC), parvalbumin interneurons (IN-PV), somatostatin interneurons (IN-SST), SV2C interneurons (IN-SV2C), VIP interneurons (IN-VIP), maturing neurons (Neu-mat), NRGN-expressing neurons (Neu-NRGN-I), NRGN-expressing neurons (Neu-NRGN-II), endothelial cells, microglia cells, and oligodendrocytes.

### Metacells building

By using SuperCell [30] to merge transcriptionally similar cells from ASD and control samples, DCRNet builds metacells of snRNA-seq data. For SuperCell, three parameters (the number of nearest neighbors, the number of the top genes, and the graining level) need to set. Empirically, the number of nearest neighbors to build KNN (K-Nearest Neighbors) network is set to 5, and the number of the top variable genes to use for dimensionality reduction is set to 2000. Since the graining level (i.e. level of size reduction between the snRNA-seq and the metacell data) is generally recommended between 10 and 50, DCRNet selects 5 graining levels (10, 20, 30, 40, and 50) and then use 4 metrics (including purity, compactness, separation, and inner normalized variance) to perform quality controls on metacells by using MetacellAnalysisToolkit [31]. Here, purity is the proportion of the most abundant cell type within the metacell, compactness is the variance of the components within the metacell, separation is the distance to the closest metacell, and inner normalized variance is the mean-normalized variance of gene expression within the metacell. Higher values of purity and separation and lower values of compactness and inner normalized variance, indicate better metacell partitioning performance. In terms of separation and inner normalized variance, larger graining levels have better metacell partitioning. However, in terms of purity and compactness, lower graining levels perform better in metacell partitioning. Based on a balanced consideration of these 4 metrics, DCRNet sets the graining level to 30 in this work.

### Trajectory inference

Given the metacell data of each data slice, DCRNet applies SCORPIUS [32] to perform linear trajectory inference on metacells. SCORPIUS reduces the dimensionality of the metacell data and returns coordinates of the metacells represented in a 2D space. Based on the identified 2D space, SCORPIUS further orders brain metacells of each data slice along a linear trajectory. Consequently, we have obtained 48 pseudo time-series metacell data for hidden causal inference.

### Priori information of lncRNA targets

To enhance the accuracy of dynamic lncRNA causal regulation, we apply a sequence-based method LncTar [33] to identify putative lncRNA targets. We obtain the sequences of lncRNAs and mRNAs from the Ensembl [34] database (version 115) via the biomaRt [35] R package, prioritizing the longest transcript isoform per gene. LncTar predicts lncRNA-mRNA interactions by calculating the minimum free energy of the joint secondary structure formed through base pairing between two RNA transcripts. For large-scale identification of lncRNA targets, we utilize a parallelized implementation of LncTar. In this study, we applied a normalized binding free energy (ndG) threshold of ≤ −0.1 to infer significant lncRNA-mRNA interactions. This cutoff is the default setting of LncTar, derived from the distribution of normalized free energy values among random lncRNA-mRNA pairs to control false positives, and reflects the relative stability of base pairing between RNA molecules. In total, we have obtained a list of 105 649 lncRNA-mRNA interactions as priori information of dynamic lncRNA causal regulation (Supplementary Table S1).

### Dynamic lncRNA causal regulation

Causal networks are abundant in biological processes, and the hidden nature of causality is positive, negative, or dark in complex biological systems [36, 37]. To quantify positive, negative, and dark causality in the pseudo time-series metacell data, the PC (Pattern Causality) algorithm [36, 37] is introduced for hidden causal inference.

Let *R* and *G* represent the time-series data for a lncRNA and an mRNA, respectively. Given *R* and *G*, PC creates two shadow attractors, *M_R_* and *M_G_*, respectively, by identifying the optimal pair $\left(E,\tau \right)$. Here, *E* denotes the embedding dimension of the shadow attractor, and *τ* is the time lag to construct a shadow attractor.


(1) \begin{eqnarray*} \displaystyle \begin{array}{l}R=\left\{R(1),\dots, R(L)\right\}\Rightarrow{M}_R\\[3pt] {}=\left(\begin{array}{c}r(1)=<R(1),R\left(1+\tau \right),\dots, R\left(1+\left(E-1\right)\tau \right)>\\{}r(2)=<R(2),R\left(2+\tau \right),\dots, R\left(2+\left(E-1\right)\tau \right)>\\{}\vdots \\{}r\left(L-\left(E-1\right)\tau \right)=<R\left(L-\left(E-1\right)\tau \right),R\left(L-\left(E-2\right)\tau \right),\dots, R(L)>\end{array}\right)\end{array} \end{eqnarray*}

(2) \begin{eqnarray*} \displaystyle \begin{array}{l}G=\left\{G(1),\dots, G(L)\right\}\Rightarrow{M}_G\\[3pt] {}=\left(\begin{array}{c}g(1)=<G(1),G\left(1+\tau \right),\dots, G\left(1+\left(E-1\right)\tau \right)>\\{}g(2)=<G(2),G\left(2+\tau \right),\dots, G\left(2+\left(E-1\right)\tau \right)>\\{}\vdots \\{}g\left(L-\left(E-1\right)\tau \right)=<G\left(L-\left(E-1\right)\tau \right),G\left(L-\left(E-2\right)\tau \right),\dots, G(L)>\end{array}\right)\end{array} \end{eqnarray*}

Where *L* is the length of the time-series data, *r(t)* and *g(t)* are the vectors of *M_R_* and *M_G_* respectively corresponding to the state at time *t*. Among all vectors in *M_R_* and *M_G_*, the distance matrices *D_R_* and *D_G_* are calculated as follows:


(3) \begin{eqnarray*} {D}_R=\left(\begin{array}{ccc}d\left(r(1),r(1)\right)& \cdots & d\left(r(1),r\left(L-\left(E-1\right)\tau \right)\right)\\{}\vdots & \ddots & \vdots \\{}d\left(r\left(L-\left(E-1\right)\tau \right),r(1)\right)& \cdots & d\left(r\left(L-\left(E-1\right)\tau \right),r\left(L-\left(E-1\right)\tau \right)\right)\end{array}\right) \end{eqnarray*}

(4) \begin{eqnarray*} {D}_G=\left(\begin{array}{ccc}d\left(g(1),g(1)\right)& \cdots & d\left(g(1),g\left(L-\left(E-1\right)\tau \right)\right)\\{}\vdots & \ddots & \vdots \\{}d\left(g\left(L-\left(E-1\right)\tau \right),g(1)\right)& \cdots & d\left(g\left(L-\left(E-1\right)\tau \right),g\left(L-\left(E-1\right)\tau \right)\right)\end{array}\right) \end{eqnarray*}

For each point *r*(*t*) in *M_R_*, its *E*+1 nearest neighbors $N{N}_{r(t)}$ is found. From the *E+1* nearest neighbors, the projected time indices ${t}_{r_1},{t}_{r_2},\dots, {t}_{r_{E+1}}$ by projecting ${t}_1,{t}_2,\dots, {t}_{E+1}$ahead by *h* steps are:


(5) \begin{eqnarray*} {\displaystyle \begin{array}{l}N{N}_{r(t)}= argmi{n}_{\left(E+1\right)}\left\{d\left(r(t),r(1)\right),\dots, d\left(r(t),r\left(t-\left(E-1\right)\ast \tau -h\right)\right)\right\}\\ \qquad\ \ \ {}=\left\{N{N}_{r\left({t}_1\right)},N{N}_{r\left({t}_2\right)},\dots, N{N}_{r\left({t}_{E+1}\right)}\right\}\Rightarrow{t}_1,{t}_2,\dots, {t}_{E+1}\overset{h stepsahead}{\Rightarrow}\\{}{t}_1+h,{t}_2+h,\dots, {t}_{E+1}+h={t}_{r_1},{t}_{r_2},\dots, {t}_{r_{E+1}}\end{array}} \end{eqnarray*}

The distance of the projected *E+1* nearest neighbors from *r*(*t*) and *g*(*t*):


(6) \begin{eqnarray*} {d}_{r_1}=\left(d\Big(r(t),r\left({t}_{r_1}\right)\right),{d}_{r_2}=\left(d\Big(r(t),r\left({t}_{r_2}\right)\right),\dots, {d}_{r_{E+1}}=\left(d\Big(r(t),r\left({t}_{r_{E+1}}\right)\right) \end{eqnarray*}

(7) \begin{eqnarray*} {d}_{g_1}=\left(d\Big(g(t),g\left({t}_{r_1}\right)\right),{d}_{g_2}=\left(d\Big(g(t),g\left({t}_{r_2}\right)\right),\dots, {d}_{g_{E+1}}=\left(d\Big(g(t),g\left({t}_{r_{E+1}}\right)\right) \end{eqnarray*}

The future pattern ${P}_{g\left(t+h\right)}$ of $g\left(t+h\right)$ at time *t+h* is estimated:


(8) \begin{eqnarray*} {P}_{g\left(t+h\right)}=\psi \left({S}_{g\left(t+h\right)}\right) \end{eqnarray*}

Where the mapping function $\psi \left(\cdot \right)$ is a pattern extraction function that maps the space ${S}_{g\left(t+h\right)}$ to ${P}_{g\left(t+h\right)}$, ${S}_{g\left(t+h\right)}=\sum \limits_{t_n={t}_{r_1}}^{t_{r_{E+1}}}{w}_{t_n}^g{s}_{t_n}^g$, ${w}_{t_n}^g=\frac{e^{d\left(g(t),g\left({t}_n\right)\right)}}{\sum_{t_n}{e}^{d\left(g(t),g\left({t}_n\right)\right)}}$, ${s}_{t_n}^g=\left(\frac{G\left({t}_{r_2}\right)-G\left({t}_{r_1}\right)}{G\left({t}_{r_1}\right)},\dots, \frac{G\left({t}_{r_{E+1}}\right)-G\left({t}_{r_E}\right)}{G\left({t}_{r_E}\right)}\right)$.

The current pattern ${P}_{r(t)}$ of *r(t)* at time *t* is calculated:


(9) \begin{eqnarray*} {P}_{r(t)}=\psi \left({S}_{r(t)}\right) \end{eqnarray*}

Where the mapping function $\psi \left(\cdot \right)$ is a pattern extraction function that maps the space ${S}_{r(t)}$ to${P}_{r(t)}$, ${S}_{r(t)}=\sum \limits_{t_n={t}_{r_1}}^{t_{r_{E+1}}}{w}_{t_n}^r{s}_{t_n}^r$, ${w}_{t_n}^r=\frac{e^{d\left(r(t),r\left({t}_n\right)\right)}}{\sum_{t_n}{e}^{d\left(r(t),r\left({t}_n\right)\right)}}$, ${s}_{t_n}^r=\left(\frac{R\left({t}_{r_2}\right)-R\left({t}_{r_1}\right)}{R\left({t}_{r_1}\right)},\dots, \frac{R\left({t}_{r_{E+1}}\right)-R\left({t}_{r_E}\right)}{R\left({t}_{r_E}\right)}\right)$.

The strength of pattern causality from *R* to *G* at every time step *t* is:


(10) \begin{eqnarray*} PC\left[{P}_R,{P}_G,t\right]=\mathit{\operatorname{erf}}\left(\frac{\left\Vert{S}_{g\left(t+h\right)}\right\Vert }{\left\Vert{S}_{r(t)}\right\Vert}\right) \end{eqnarray*}

Where $\big\Vert{S}_{g\big(t+h\big)}\big\Vert$ and $\big\Vert{S}_{r(t)}\big\Vert$ represent the norms of ${S}_{g\left(t+h\right)}$ and ${S}_{r(t)}$, respectively, the error function $\mathit{\operatorname{erf}}(x)=\frac{1}{\sqrt{\pi }}\int_{-x}^x{e}^{-{t}^2} dt$.

At time *t*, positive, negative, and dark causality are represented as *P*(*t*), *N*(*t*), and *D*(*t*), respectively. Since multiple causality types cannot coexist, only one type of causal pattern exists at a time *t*. The causality type with the largest causal strength is regarded as the final causal pattern from *R* to *G*.

In this work, DCRNet utilizes patterncausality [38] R package to calculate positive, negative, and dark causality between lncRNAs and mRNAs. When using the patterncausality tool, the embedding dimension *E* is set to 3, the time lag *τ* is 1, the distance metric used is Euclidean, and the number of steps ahead *h* is 1. To accelerate the speed of hidden causal inference, DCRNet uses parallel computing with 48 CPU cores to infer positive, negative, and dark causality between lncRNAs and mRNAs. Following the pattern causality framework [36, 37], a dominant strength above 0.60 ensures that the assigned causal mode accounts for a clear majority of the total causal signal, thus retaining only those regulatory relationships supported by a strong and unambiguous causal pattern. Therefore, for identifying significant lncRNA-mRNA causal relationships, the cutoff of positive, negative, or dark causal strength is set to 0.60. To assess the robustness of this threshold choice, we perform sensitivity analyses across thresholds of 0.50, 0.55, 0.60, 0.65, and 0.70. Across all thresholds examined, dark causality consistently remains the most prevalent causal type. The threshold of 0.60 is selected as it provides a balanced trade-off between retaining biologically meaningful regulatory signals and minimizing potential false positives. The causality type with the largest causal strength is regarded as the final causal pattern from an lncRNA to an mRNA. Given 48 pseudo time-series metacell data spanning 5 brain biological contexts (i.e. diagnosis, region, age, sex, and cell type), DCRNet constructs 48 lncRNA causal regulatory networks in total.

### Heterogeneity analysis

Given two lncRNA causal regulatory networks $Ne{t}_i$ and $Ne{t}_j$, the heterogeneity $Het\left( Ne{t}_i, Ne{t}_j\right)$ between $Ne{t}_i$ and $Ne{t}_j$ is defined as:


(11) \begin{eqnarray*} Het\left( Ne{t}_i, Ne{t}_j\right)=1-\frac{\mid Ne{t}_i\cap Ne{t}_j\mid }{\mathit{\min}\left(| Ne{t}_i|,| Ne{t}_j|\right)} \end{eqnarray*}

Where ${\mid} Ne{t}_i\cap Ne{t}_j{\mid}$ is the number of common lncRNA-mRNA causal relationships between $Ne{t}_i$ and $Ne{t}_j$, $\mathit{\min}\left(| Ne{t}_i|,| Ne{t}_j|\right)$ denotes the number of lncRNA-mRNA causal relationships in the smallest network between $Ne{t}_i$ and $Ne{t}_j$. $Het\left( Ne{t}_i, Ne{t}_j\right)$ ranges from 0 to 1. A higher value of $Het\left( Ne{t}_i, Ne{t}_j\right)$ implies that $Ne{t}_i$ and $Ne{t}_j$ have greater heterogeneity with each other. In terms of lncRNA causal regulatory networks, we use $Het\left( Ne{t}_i, Ne{t}_j\right)$ to evaluate the heterogeneity between different brain biological contexts (i.e. diagnosis, region, age, sex, and cell type).

### ASD susceptibility lncRNAs identification

Differential expression analysis is commonly employed to identify ASD susceptibility lncRNAs. However, for single-cell RNA sequencing data, differential expression analysis is challenged by excessive zeros, normalization, donor effects, and cumulative biases [39]. Additionally, differential expression analysis focuses on lncRNA expression levels and ignores their downstream targets. To address these limitations, we develop a network-based approach to identify ASD susceptibility lncRNAs by analyzing differential causal networks between ASD and control. For each lncRNA, we quantify network rewiring by calculating the total number of rewired causal edges (i.e., sum of gained and lost edges). The susceptibility score (*SS*) and significance *P*-value for each lncRNA *i* is computed as follows.


(12) \begin{eqnarray*} {\displaystyle \begin{array}{l}S{S}_i=\frac{Nu{m}_i-\mu }{\sigma}\\{}{p}_i=1-\phi \left(R{S}_i\right)\end{array}} \end{eqnarray*}

Where $Nu{m}_i$ is the number of rewired causal edges for lncRNA *i*, *μ* represents the mean number of rewired causal edges across all lncRNAs, *σ* denotes the standard deviation, and *ϕ*is the cumulative distribution function (CDF) of the standard normal distribution.

We define lncRNAs with statistically significant susceptibility scores (adjusted *P* < .05, adjusted by the Benjamini–Hochberg method [40]) as ASD susceptibility lncRNAs, characterized by markedly disrupted causal regulatory patterns in ASD.

### Potential lncRNA biomarkers prediction

Hub lncRNAs are highly connected nodes in lncRNA causal regulatory networks and are often regarded as promising biomarkers. Here, we identify hub lncRNAs by applying the cumulative Poisson distribution probability:


(13) \begin{eqnarray*} p\left(y\ge x\right)=1-\sum \limits_{i=0}^{x-1}\frac{\lambda^i{e}^{-\lambda }}{i!} \end{eqnarray*}

where *λ = np*, $p=\frac{A}{B}$, *n* is the number of genes (including lncRNAs and mRNAs), *A* is the number of lncRNA-mRNA causal relationships in a lncRNA causal regulatory network, and *B* is the number of all possible lncRNA-mRNA causal pairs. Smaller *P*-value of an lncRNA indicates that the lncRNA is more likely to be a hub. Here, the cutoff of the *P*-value is set to .05.

In this work, biomarkers for cell type and sex are each classified into two categories: specific and enhanced. Specifically, hub lncRNAs identified exclusively in a single cell type are designated as cell type-specific biomarkers, and those identified exclusively in a single sex are designated as sex-specific biomarkers. By contrast, hub lncRNAs with a degree at least two-fold higher in a specific cell type than the average across all other cell types are classified as cell-type-enhanced biomarkers, and those with a degree at least two-fold higher in a specific sex than that in the other sex are classified as sex-enhanced biomarkers, following the paradigm established for cellularly-elevated lncRNAs [41].

### Immune-related lncRNA detection

Among 17 major cell types in the brain, microglia are the resident immune cells of the central nervous system and play a critical role in the pathogenesis of ASD [42, 43]. By modulating gene expression in immune cells such as microglia, lncRNAs play crucial regulatory roles in the neuroimmune system [44]. Similar to the identification of lncRNA biomarkers, hub lncRNAs are also identified using the cumulative Poisson distribution probability. In this work, we define that hub lncRNAs specifically identified in microglia under either ASD or control conditions are context-specific immune lncRNAs. Additionally, a hub lncRNA identified in microglia is also considered as an immune lncRNA (i.e. a context-enhanced immune lncRNA) if its degree in ASD is at least two-fold higher or lower than its degree under control condition.

### Functional enrichment analysis

We conduct functional enrichment analysis using clusterProfiler [45] to assess the involvement of ASD susceptibility lncRNAs in the pathogenesis of ASD and the implication of immune-related lncRNAs in the immune regulation. Functional enrichment analysis is conducted for each ASD susceptibility lncRNA using its rewired targets between ASD and control, whereas for each immune-related lncRNA, both rewired and shared targets between ASD and control are used. In this work, the functional enrichment analysis cover Gene Ontology (GO) [46] including Biological Process (BP), Molecular Function (MF), and Cellular Component (CC), as well as the Kyoto Encyclopedia of Genes and Genomes (KEGG) [47] and Reactome [48] pathways. For the targets of each lncRNA, terms with adjusted *P* < .05 (Benjamini–Hochberg correction) are considered significantly enriched.

### Classification analysis

To evaluate the diagnostic performance of the identified lncRNA biomarkers, we employ a classification analysis using Support Vector Machine (SVM) [49] implemented in the e1071 [50] R package. Separate classification tasks are performed for binary (sex) and multiclass (cell type) predictions within both ASD and control cohorts. The discriminative capacity of each model is assessed using 10-fold cross-validation. Model performance for both binary and multiclass classification is evaluated using three standard metrics: Accuracy, Area Under the receiver operating characteristic Curve (AUC), and F1-score. Here, Accuracy is the proportion of correct predictions among the total number of cases, AUC measures the overall ability of a model to distinguish between classes across all classification thresholds, and F1-score is the harmonic mean of precision and recall. Higher values of Accuracy, AUC, and F1-score indicate better classification performance.

### Cell–cell interaction network construction

In this work, we integrate predicted lncRNA causal regulatory networks with putative ligand–receptor interaction networks to assess intercellular interaction strength between different cell types. The ligand–receptor interactions are compiled from five databases: CellPhoneDB [51], CellChatDB [52], CellTalkDB [53], Cellinker [54], and CellCall [55]. We retain only those ligand–receptor pairs involving mRNAs present in the predicted lncRNA causal networks for each cell type. In terms of the combined networks ($C{N}_i$ and $C{N}_j$ for cell types *i* and *j*, respectively), the interaction strength $IS\left(i,j\right)$ between the two cell types is calculated as:


(14) \begin{eqnarray*} IS\left(i,j\right)=\frac{\mid C{N}_i\cap C{N}_j\mid }{\mathit{\min}\left(|C{N}_i|,|C{N}_j|\right)} \end{eqnarray*}

Where ${\mid} C{N}_i\cap C{N}_j{\mid}$ indicates the number of shared interactions between $C{N}_i$ and $C{N}_j$, $\mathit{\min}\left(|C{N}_i|,|C{N}_j|\right)$ represents the number of interactions in the minimum network between $C{N}_i$ and $C{N}_j$. The value of $IS\left(i,j\right)$ ranges from 0 to 1, where 0 indicates no interaction and 1 indicates perfect interaction.

### Comparison analysis

In this work, we also apply Cycle [25], Granger causality [56], and seqICPnl (Sequential Non-linear Invariant Causal Prediction) [57] to infer lncRNA causal regulatory networks. For both sequential causality and correlation-based methods, a significance threshold of *P* < .01 is applied to infer regulatory relationships. For validation, the ground truth of lncRNA-mRNA interactions are acquired from RegNetwork [58], and we only retain the interactions with high or medium confidence scores. Method performance is evaluated based on the percentage of causal predictions that are confirmed by the limited ground truth, and a higher percentage of validated predictions (true positives) indicate superior performance.

We independently apply DCRNet, Cycle, Granger, and seqICPnl to perform differential causal network analysis between ASD and control, identifying ASD susceptibility lncRNAs. The identified ASD susceptibility lncRNAs are then cross-referenced with cell type-specific differential expression results from an independent snRNA-seq dataset [59, 60], where validation is defined as differential expression in at least one cell type between ASD and control. The validation rate serves as the primary metric to compare the efficiency of the three methods.

To further benchmark the biological relevance of the inferred regulatory pairs, we evaluate their overlap with differentially expressed genes (DEGs) from the same snRNA-seq dataset [28]. We consider two independent DEG sets: cell type-specific and region-dependent. For each method, we map the ASD-associated rewired pairs to the cell type-specific DEGs and the region-dependent rewired pairs to the region-dependent DEGs. The proportion of DEGs covered by at least one rewired lncRNA-mRNA pair is then calculated for each method to assess functional relevance.

### Method execution

Each execution of DCRNet on single-nucleus RNA sequencing data is performed in a separate task. Each task is allocated 48 CPU cores of Intel(R) Xeon(R) Platinum 8375C CPU at 2.90 GHz, and one R session is opened for each task.

## Results

### Dynamic causal regulation network

We introduce DCRNet, a hidden causality approach to elucidate dynamic lncRNA causal regulation in ASD from snRNA-seq data (Fig. 1). According to the diagnosis (ASD and control, 2 data slices), region (ACC and PFC, 4 data slices), age (<18 and > =18, 4 data slices), sex (Male and Female, 4 data slices), and cell type (17 cell types, 34 data slices) of brain cells, DCRNet divides the snRNA-seq data into 48 data slices (Supplementary Table S2). Since most of highly similar brain cells correspond to technical noise and not to biologically relevant heterogeneity, DCRNet builds metacells to remove technical noise while preserving the biological information of each data slice. Here, metacells are disjoint homogeneous groups of highly similar cells [31]. To investigate state changes in physiological and pathological conditions, DCRNet further uses trajectory inference to order brain metacells of each data slice along a linear trajectory. To explore dynamic causal regulation by lncRNAs, DCRNet employs hidden causal inference to identify causal relationships between lncRNAs and mRNAs in each data slice. Finally, DCRNet performs heterogeneity analysis to delineate dynamic lncRNA causal regulation across diverse brain biological contexts (i.e. diagnosis, region, age, sex, and cell type).

**Figure 1:**
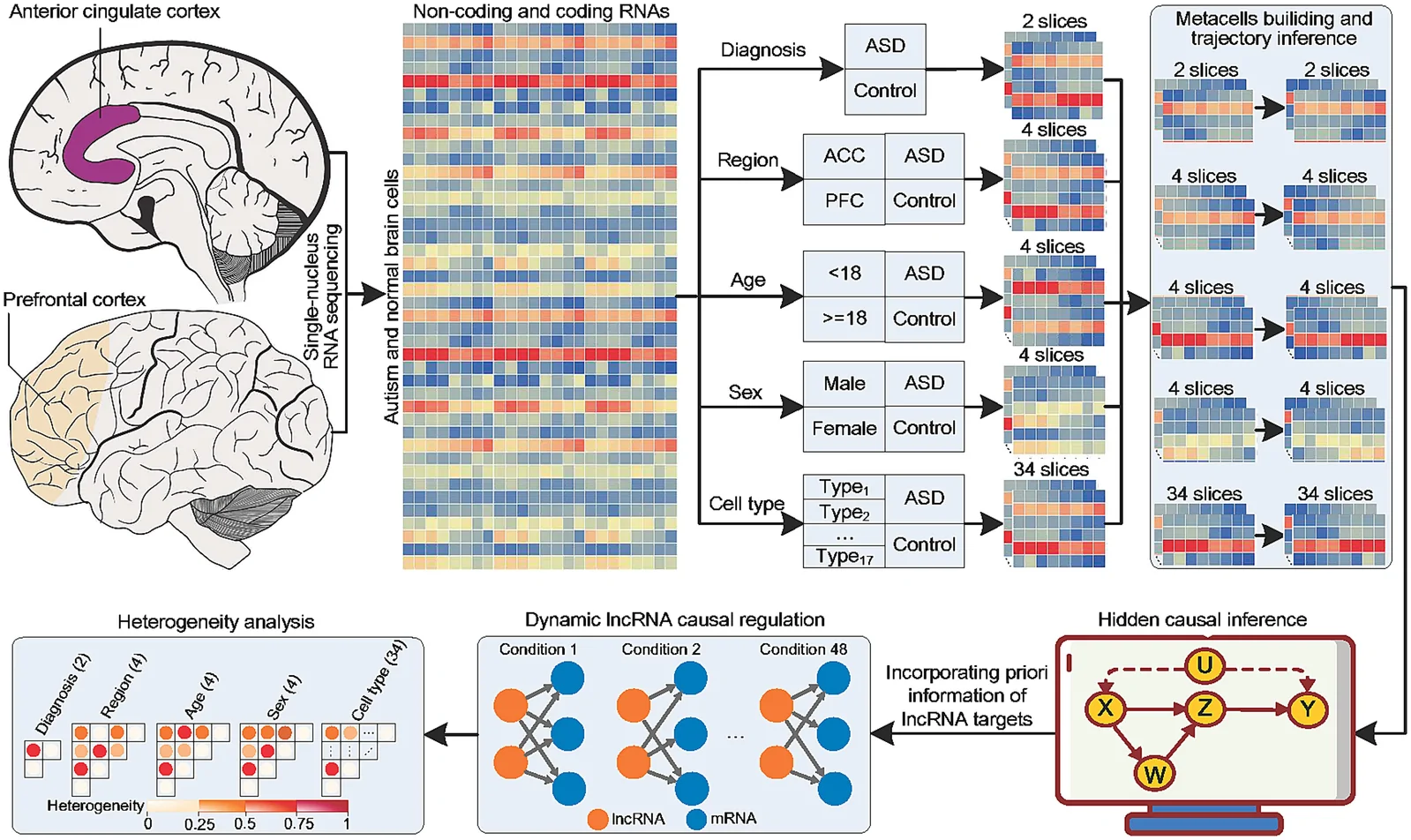
Schematic workflow of DCRNet. According to brain biological contexts (i.e. diagnosis, region, age, sex, and cell type), the snRNA-seq data of autism and control brain cells is splitted into 48 data slices. For each data slice, metacells are built for removing technical noise and preserving the biological information of it. For the identified metacells, DCRNet uses trajectory inference to order them along a linear trajectory. Given the processed 48 data slices, DCRNet applies hidden causal inference and incorporates priori information of lncRNA targets to infer dynamic lncRNA causal regulation. Based on the identified dynamic lncRNA causal regulation, DCRNet further conducts heterogeneity analysis to capture conditional shifts in lncRNA regulation.

### Dark causality is the most frequently observed type of lncRNA regulation across the brain biological contexts

Causality is an intrinsic, structural property of biological networks, governing the fundamental landscape of a biological system [61]. To characterize the spectrum of lncRNA causal regulation across diverse brain biological contexts including diagnosis, region, age, sex, and cell type, we quantify the prevalence of three distinct causal types: positive, negative, and dark causality. We find that dark causality, a hybrid form of positive and negative interdependencies, is the most frequently observed causal type across all biological contexts examined, and this pattern holds consistently regardless of whether prior information is incorporated into the causal inference (Fig. 2, Supplementary Tables S3 and S4). To assess whether the counts of the three causal types differ between ASD and control groups, we perform paired Wilcoxon signed-rank tests for each biological context, with *P*-values adjusted using the Benjamini–Hochberg correction. No statistically significant differences are observed between ASD and control groups in any of the five contexts (all adjusted *P* > .05), regardless of prior information. The consistent prevalence of dark causality across diagnosis, region, age, sex, and cell type suggests that it is a common mode of lncRNA regulation in ASD, indicating that lncRNA-mediated regulation frequently operates through complex, blended influences rather than purely positive or negative effects.

**Figure 2:**
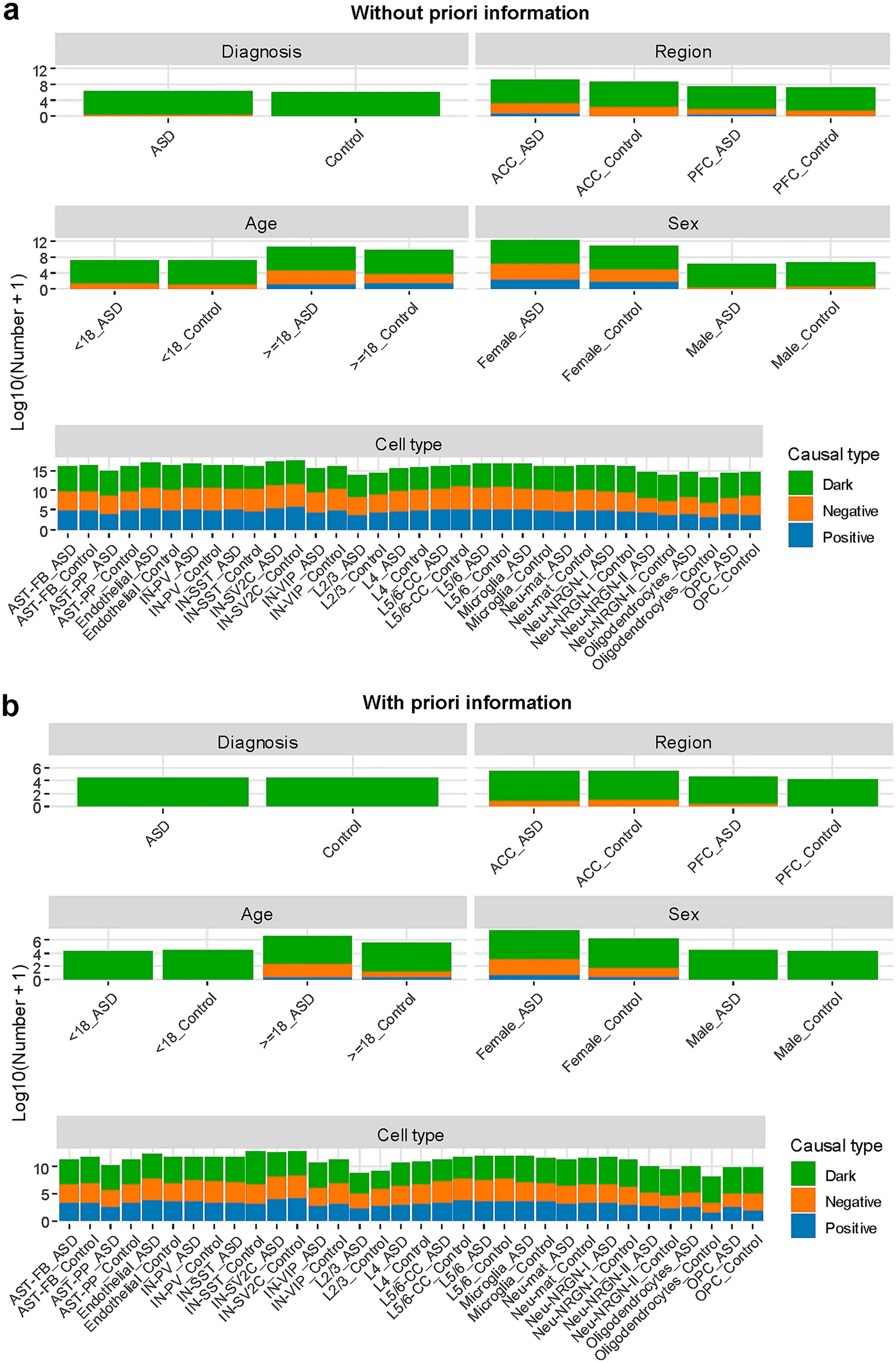
The spectrum of lncRNA causal regulation across diverse brain biological contexts. Number of positive, negative, and dark causality (a) without and (b) with priori information in diverse brain biological contexts including diagnosis, region, age, sex, and cell type.

### Dynamic lncRNA causal regulation emerges in diverse brain biological contexts

It is demonstrated that lncRNAs exhibit dynamic and context-dependent regulatory roles in ASD, contributing to the disorder’s etiological complexity [28, 62]. To investigate the dynamic nature of lncRNA-mediated regulation, we analyze the scale and heterogeneity of lncRNA causal regulatory networks across diverse brain biological contexts, including diagnosis, region, age, sex, and cell type (Fig. 3). For diagnosis, the network is expanded in ASD compared to control, with a heterogeneity score of 0.40, indicating both increased connectivity and structural rewiring of regulatory interactions (Fig. 3a). Between brain regions, the number of regulatory relationships differs notably, with heterogeneity scores >0.30 between ACC and PFC, reflecting spatial differences in network connectivity (Fig. 3b). In terms of age, we observe a notable shift in the quantity of regulatory interactions between donors under and over 18 years old, with heterogeneity scores exceeding 0.31, suggesting developmental stage-dependent differences in network architecture (Fig. 3c). For sex, sex-dimorphic differences are observed in both the number of causal interactions and heterogeneity scores, reinforcing sex as an important source of regulatory variation (Fig. 3d). At the cell-type level, notable differences are observed in both the number and heterogeneity of causal relationships, with immune cells such as Microglia in ASD showing relatively high connectivity and distinct regulatory patterns (Fig. 3e). Together, these results suggest that lncRNA causal regulatory networks exhibit dynamic organization in a multilayered, context-dependent manner, varying in both scale and structural heterogeneity across the biological contexts examined.

**Figure 3:**
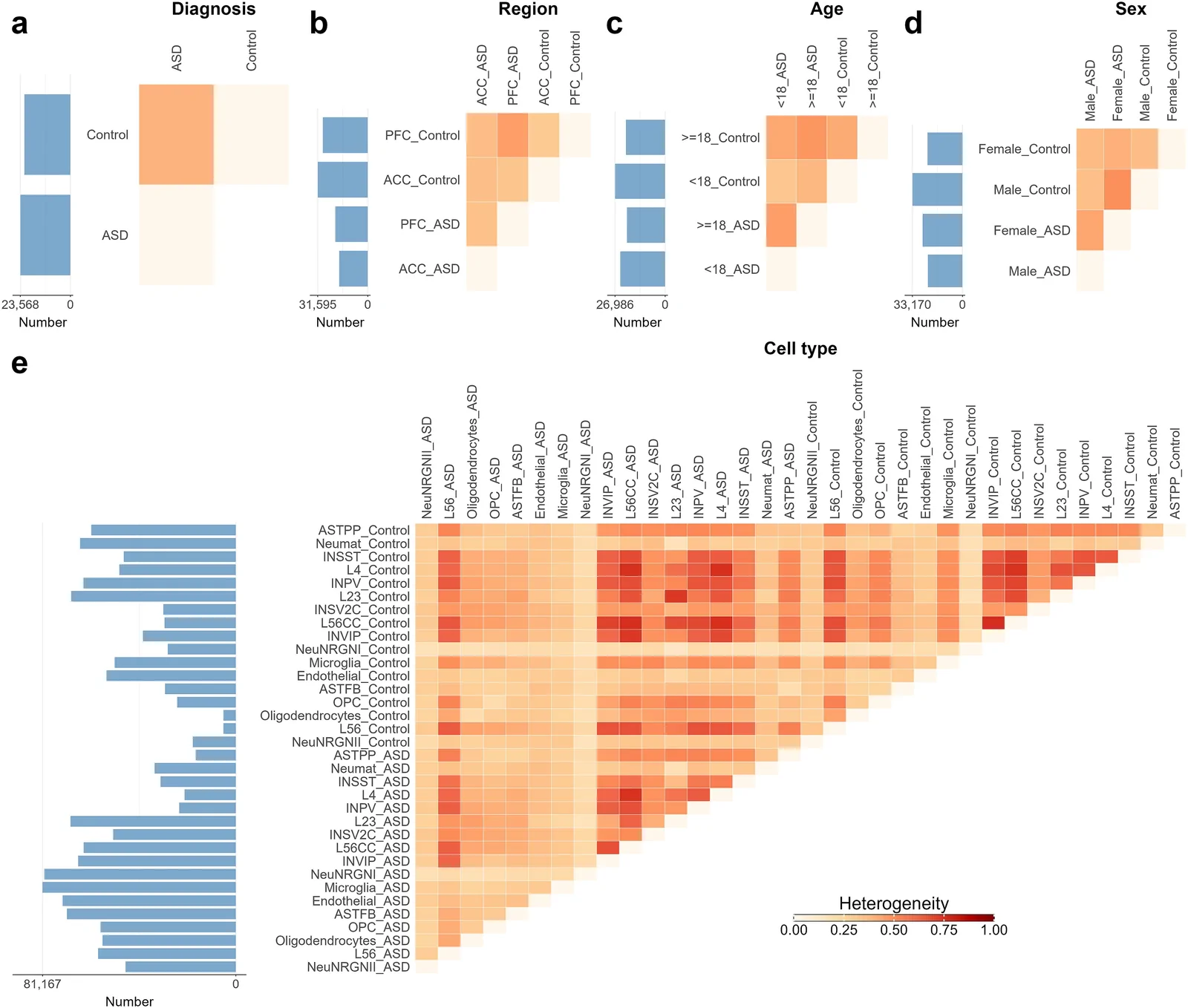
The scale and heterogeneity of lncRNA causal regulation. The lncRNA causal regulation across diverse brain biological contexts, including (a) diagnosis, (b) region, (c) age, (d) sex, and (e) cell type. The bar plots show the number of lncRNA-mRNA causal relationships in each condition, and the heat maps indicate the heterogeneity score of lncRNA causal networks between conditions in each biological context.

### ASD susceptibility lncRNAs are significantly involved in the pathogenesis of ASD

In ASD, large-scale genetic and transcriptomic studies have revealed widespread dysregulation of lncRNA expression and highlighted their genomic loci as enriched for ASD-associated susceptibility variants [62, 63]. To systematically investigate the potential role of lncRNAs in the molecular etiology of ASD, we perform differential causal network analysis between ASD and control, which lead to the identification of eight ASD susceptibility lncRNAs (*TRMT2B-AS1, MFSD12-AS1, LINC00903, EPC1-AS1, LINC01632, LINC01840, P3H2-AS1, HRG-AS1*) exhibiting statistically significant susceptibility scores (Fig. 4a, Supplementary Table S5). We further compare the eight ASD susceptibility lncRNAs with cell type-specific differential expression results from an independent snRNA-seq dataset that profiles the same cortical cell types [59, 60]. Four of the eight lncRNAs (*LINC00903, LINC01632, LINC01840, P3H2-AS1*) are independently identified as differentially expressed in at least one cell type in ASD versus control (adjusted *P* < .05, adjusted by the Benjamini–Hochberg method [40]), providing orthogonal support for their potential involvement in ASD.

**Figure 4:**
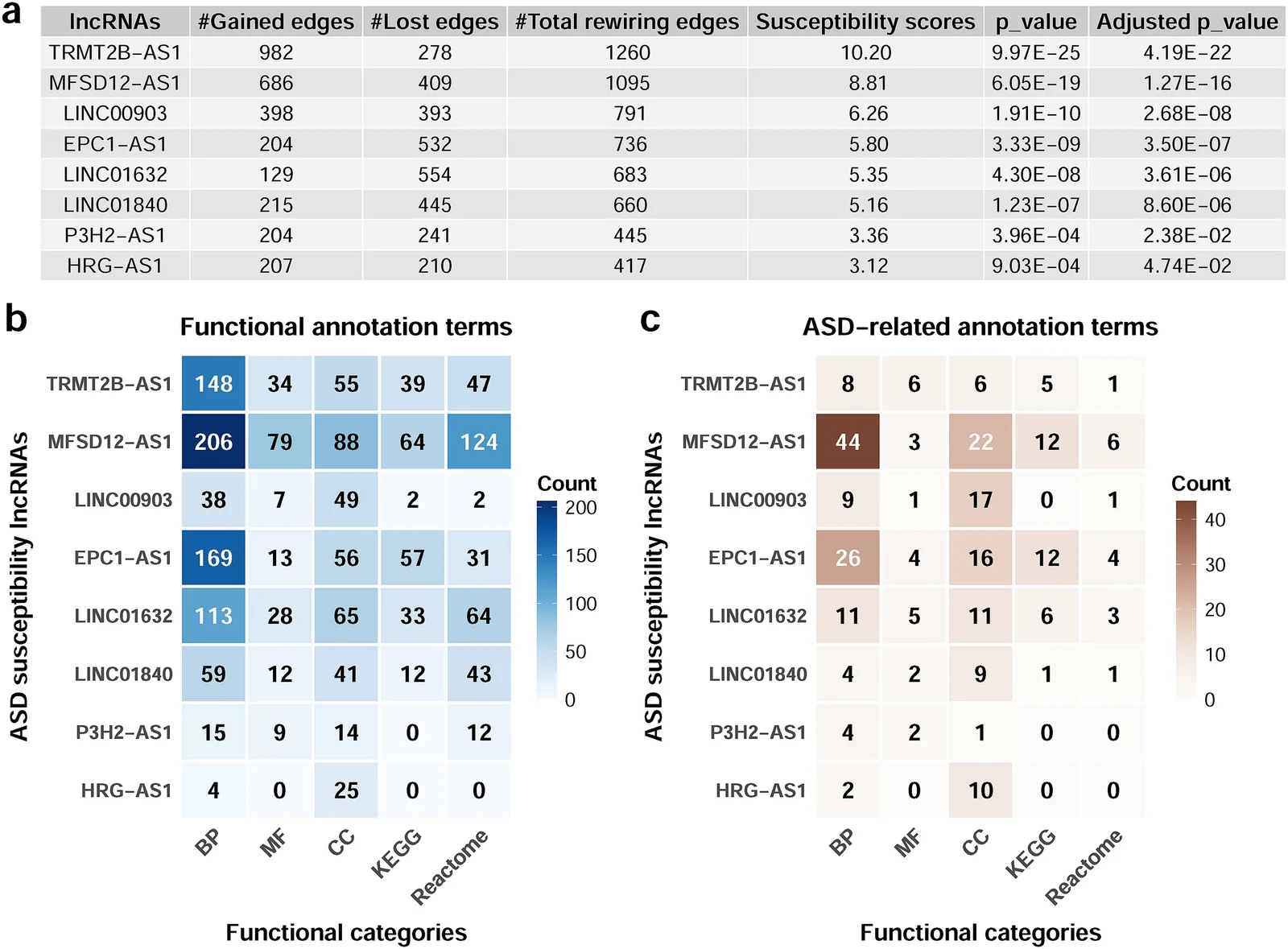
Functional enrichment analysis of eight ASD susceptibility lncRNAs. (a) Susceptibility scores of eight ASD susceptibility lncRNAs, (b) heat map of functional annotation terms of eight ASD susceptibility lncRNAs, and (c) heat map of ASD-related annotation terms of eight ASD susceptibility lncRNAs.

As illustrated in Fig. 4b, the eight ASD susceptibility lncRNAs exhibit broad enrichment across multiple functional categories, including BP, MF, CC, KEGG, and Reactome terms (Supplementary Tables S6–S10). Among the eight ASD susceptibility lncRNAs examined, *TRMT2B-AS1, MFSD12-AS1, EPC1-AS1*, and *LINC00903* show the highest degree of enrichment for functional annotation terms. Moreover, all eight identified ASD susceptibility lncRNAs are enriched in at least one functional term associated with neurodevelopment and synaptic function, both of which are known to underlie ASD pathogenesis (Fig. 4c). Collectively, these results suggest that the identified lncRNAs may contribute to the molecular mechanisms underlying ASD and could represent candidates for further mechanistic investigation.

### Potential lncRNA biomarkers can distinguish sex and cell types

Sex and cell type are two fundamental axes of biological heterogeneity in ASD [5, 64]. Sex differences are crucial for understanding ASD prevalence, while cell type-specific molecular disruptions are central to ASD’s neurodevelopmental pathology. Therefore, investigating potential lncRNA biomarkers across sex and cell type can reveal context-specific pathobiology and inform precise diagnostic strategies.

We identify distinct profiles of lncRNA biomarkers in ASD versus control contexts (Fig. 5a, Supplementary Table S11). For sex, a greater number of context-specific biomarkers (81) is found in ASD than context-enhanced ones (48), and a similar pattern is observed in control (128 versus 56). In contrast, for cell type, context-enhanced biomarkers vastly outnumber context-specific ones in both ASD (268 versus 27) and control (328 versus 22). This divergence suggests that the biological underpinnings of ASD differ fundamentally across these two axes: sex effects may reflect more stable, developmental programming differences, whereas cell-type disruptions may indicate active, disease-progress-related alterations in specific cellular environments.

**Figure 5:**
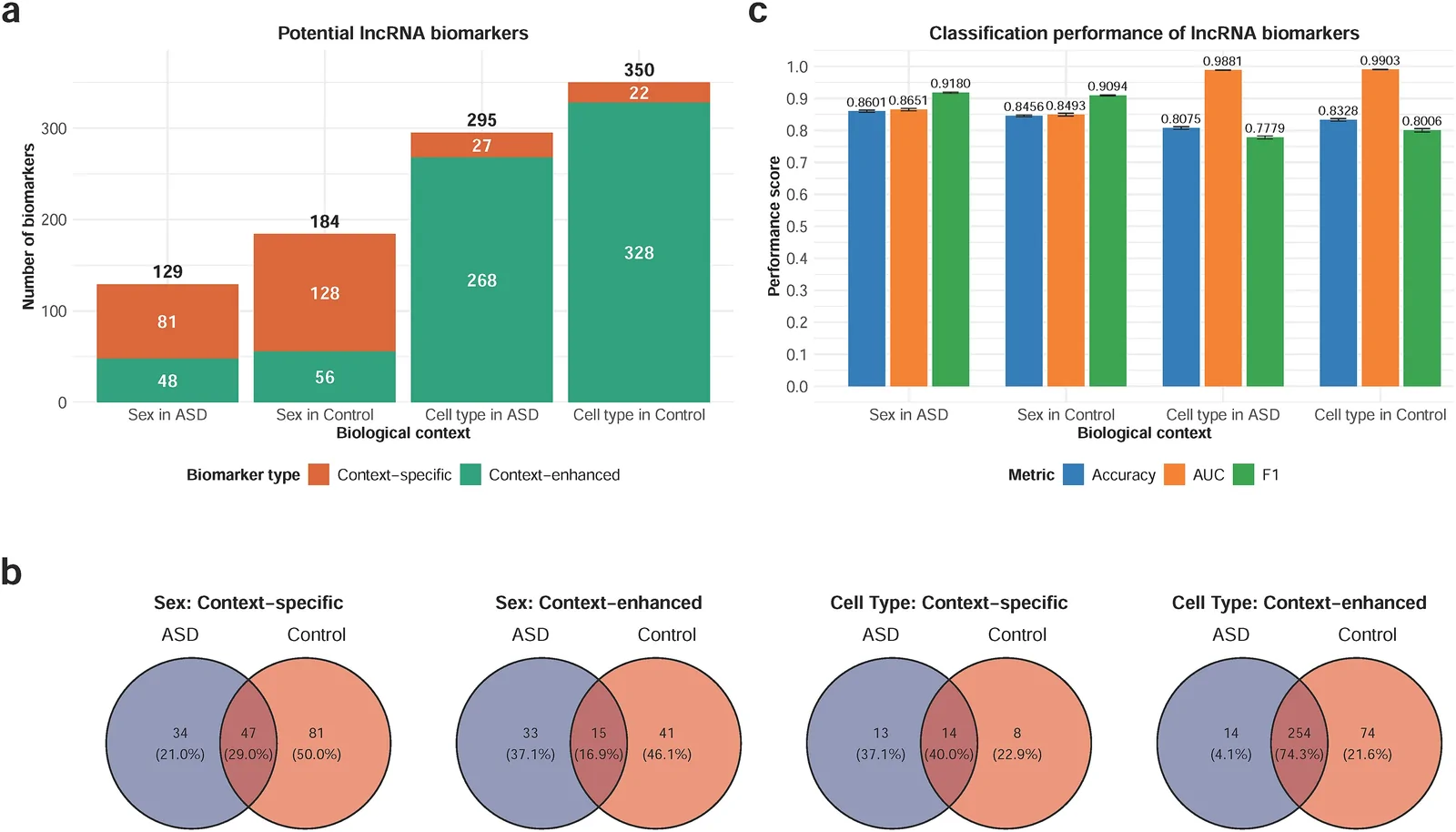
Potential lncRNA biomarkers. (a) Number of lncRNA biomarkers. (b) Venn diagrams of lncRNA biomarkers of sex and cell type. (c) Classification performance of lncRNA biomarkers in distinguishing sex and cell types.

A comparative analysis of shared lncRNA biomarkers between ASD and control reveals distinct patterns of overlap across sex and cell type (Fig. 5b). For sex, only a limited subset of context-specific (29.0%) and context-enhanced (16.9%) biomarkers are common between ASD and control. The small common set may suggest that ASD substantially alters or employs different sex-biased lncRNA regulatory networks compared to control, pointing to a more pronounced rewiring of sex-specific molecular pathways in the disorder. In contrast, for cell type, a higher proportion of context-specific (40.0%) and context-enhanced (74.3%) are shared between ASD and control. This result suggests that cellular lncRNA regulatory architecture is broadly shared between ASD and control, with ASD-associated dysregulation potentially involving the enhancement of pre-existing cell type-specific programs.

The classification performance of lncRNA biomarkers is evaluated using Accuracy, AUC, and F1, with 95% confidence intervals (CI) derived from 10-fold cross-validation (Fig. 5c). In distinguishing sex within ASD, the lncRNA biomarker set achieves an Accuracy of 0.8601 (95% CI: 0.8567–0.8635), AUC of 0.8651 (95% CI: 0.8612–0.8691), and F1 score of 0.9180 (95% CI: 0.9161–0.9199). For cell type classification in ASD, performance reaches an Accuracy of 0.8075 (95% CI: 0.8032–0.8118), AUC of 0.9881 (95% CI: 0.9876–0.9887), and F1 score of 0.7779 (95% CI: 0.7730–0.7826). Comparative analysis across contexts confirms consistently high discriminatory power, with all Accuracy, AUC, and F1 scores exceeding 0.80 for control conditions. Specifically, for sex in control, Accuracy is 0.8456 (95% CI: 0.8429–0.8483), AUC is 0.8493 (95% CI: 0.8449–0.8537), and F1 is 0.9094 (95% CI: 0.9078–0.9109). For cell type in control, Accuracy is 0.8328 (95% CI: 0.8287–0.8369), AUC is 0.9903 (95% CI: 0.9900–0.9906), and F1 is 0.8006 (95% CI: 0.7950–0.8059). These results suggest that lncRNA biomarkers can effectively distinguish sex and cell types in ASD and control contexts, highlighting their potential utility for further investigation.

### Immune-related lncRNAs are implicated in immune regulation

Growing evidence implicates immune dysregulation in the pathophysiology of ASD, linking neuroinflammation and altered immune signaling to behavioral and synaptic anomalies [65–68]. As an important layer of gene regulation, lncRNAs have been increasingly implicated in immune responses and neurodevelopmental processes [62, 69]. To investigate their potential role in ASD-related immune dysregulation, we identify 2 functionally distinct classes of immune-associated lncRNAs, including 29 context-specific and 11 context-enhanced lncRNAs. We further perform functional enrichment analysis to systematically characterize their involvement in immune regulation (Supplementary Tables S12–S16).

Functional enrichment analysis shows that 26 out of 29 context-specific lncRNAs are significantly associated with at least one BP, MF, CC, KEGG, or Reactome functional term (Fig. 6a), and 15 lncRNAs (*AQP4-AS1, ATP6V0D1-DT, CBR3-AS1, DISC1FP1, FAM182B, HCG17, KCNQ1OT1, LINC00486, LINC01090, LINC02197, LY6E-DT, MAPT-AS1, MIR137HG, PRDM6-AS1, SNHG16*) are enriched in at least one functional term directly linked to immune regulation (Fig. 6b). Likewise, all 11 context-enhanced lncRNAs are significantly enriched in at least one functional term (Fig. 6c), and four lncRNAs (*MAPKAPK5-AS1, MIR4300HG, UGDH-AS1, MAST3-AS1*) are involved in at least one functional term related to immune regulation (Fig. 6d). Additionally, previous studies support roles for two context-specific lncRNAs (*KCNQ1OT1* and *NEAT1*) in immune modulation [70, 71]. Collectively, our findings suggest that the identified 20 immune-related lncRNAs (16 context-specific and 4 context-enhanced immune lncRNAs) are implicated in immune regulation of ASD and may serve as candidates for further immune-related exploration.

**Figure 6:**
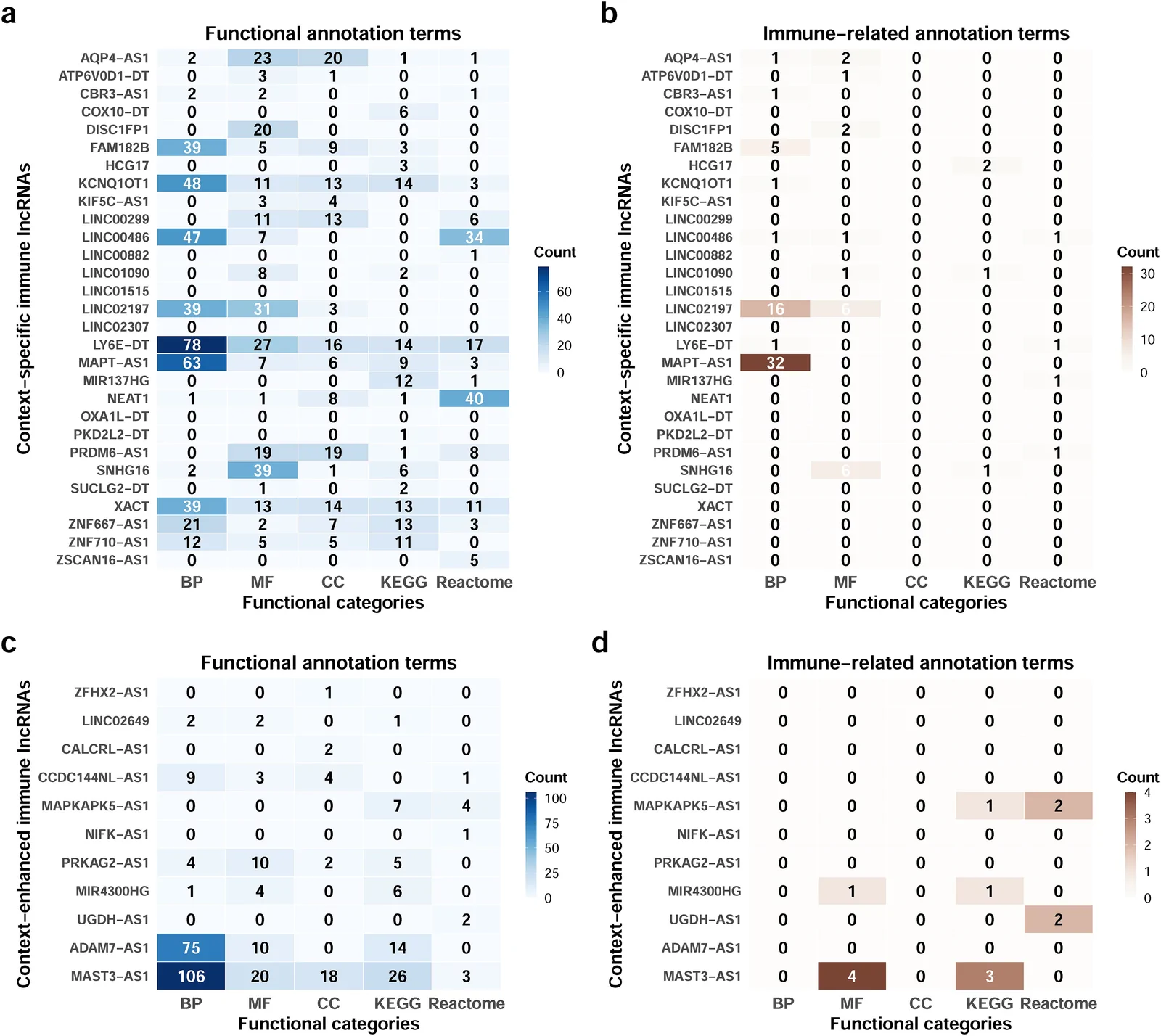
Functional enrichment analysis of 29 context-specific and 11 context-enhanced immune lncRNAs. (a) Heat map of functional annotation terms of 29 context-specific immune lncRNAs, (b) heat map of immune-related annotation terms of 29 context-specific immune lncRNAs, (c) heat map of functional annotation terms of 11 context-enhanced immune lncRNAs, and (d) heat map of immune-related annotation terms of 11 context-enhanced immune lncRNAs.

### Cell–cell interaction networks diverge between ASD and control cohorts

Dysregulation of cell–cell interaction networks represents a central pathological feature in ASD, reflecting altered neuroimmune crosstalk, glial support, and neuronal circuit coordination [28]. The comparative analysis of cell–cell interaction networks between ASD and control cortical tissues reveals reorganization of intercellular signaling [72]. To map these alterations, we integrate predicted lncRNA causal networks with putative ligand–receptor interactions to systematically construct cell–cell interaction networks for both ASD and control contexts (Supplementary Tables S17 and S18). This indirect, inference-based reconstruction provides a systematic view of potential preliminary communication differences.

In the ASD network, interactions involving Microglia show higher strength with multiple neuronal cell types (e.g. L23, INPV, L4) and other glial cells (e.g. ASTFB, ASTPP, OPC, Oligodendrocytes), suggesting possible neuroimmune crosstalk (Fig. 7a). Similarly, OPC and Oligodendrocytes exhibit enhanced connectivity, which may relate to myelination or glial-neuronal support. In contrast, the control network displays more balanced and spatially structured interactions, with stronger engagement of ASTFB, NeuNRGNI, and NeuNRGNII cell subtypes, which may reflect homeostatic astrocyte-neuron coupling (Fig. 7b).

**Figure 7:**
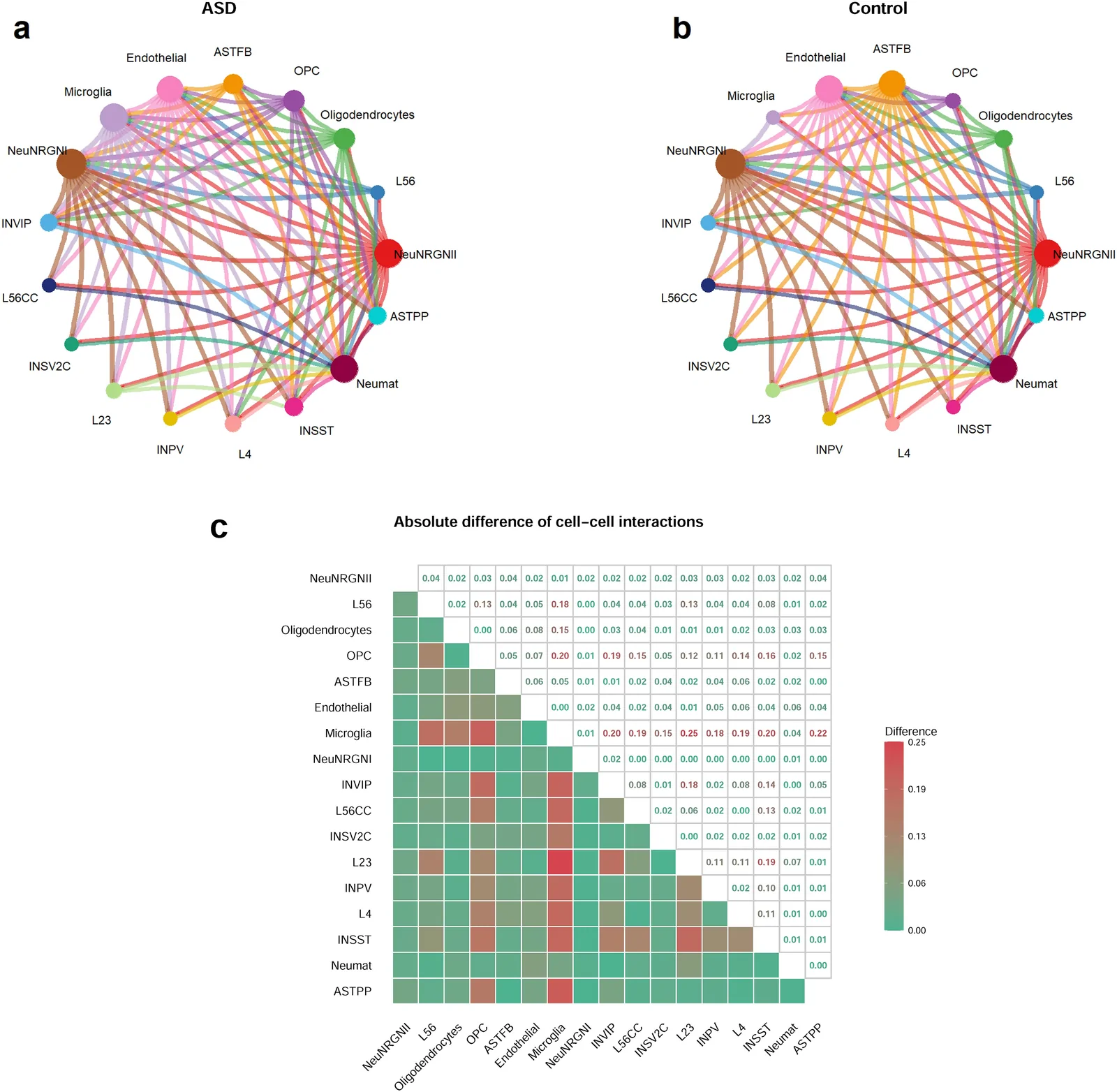
Cell–cell interactions in ASD and control cohorts. (a) Visualization of cell–cell interactions with a strength above a threshold of 0.60 in ASD. (b) Visualization of cell–cell interactions with a strength above a threshold of 0.60 in control. (c) Absolute difference of cell–cell interactions between ASD and control cohorts. Node colors represent different cell types, and edges indicate the presence of interactions between cell types.

As shown in Fig. 7c, 20.59% of all cell–cell interactions (28 of 136) are altered between ASD and control networks, with an absolute difference exceeding 0.10. Notably, Microglia show preferential connectivity with several neuronal types (e.g. L23, INPV, L4) and interneurons (e.g. INVIP, INSV2C), consistent with the notion of immune-related synaptic modulation in ASD [73]. Furthermore, OPC interactions are broadly intensified, paralleling the evidence of white matter and oligodendrocyte dysfunction in ASD [4, 74]. Subtle but consistent reductions are observed in ASTPP interactions with specific neuronal cell types, particularly inhibitory interneurons (e.g. INPV, INSST) and excitatory neurons in key cortical layers (e.g. L23, L4), suggesting a possible contribution to astrocytic modulation of synaptic function and network integration. These findings imply that ASD pathophysiology may involve system-level alterations in intercellular communication rather than isolated cellular deficits. The observed divergence in interaction strength, particularly involving Microglia and OPC, highlights these cell types as potential key nodes in ASD-associated network changes.

### In comparison to correlation-based and sequential causality methods

We evaluate the performance of DCRNet against Cycle [25], Granger [56], and seqICPnl [57] across 48 distinct brain biological conditions (Supplementary Table S19). As shown in Fig. 8a, both DCRNet and seqICPnl achieve the top rank in 32.35% (11 of 34) of the cell type-specific conditions, indicating comparable performance in inferring lncRNA-mediated regulatory links within individual cell types. In contrast, Granger achieves the highest rank primarily in 35.71% (5 of 14) of conditions related to diagnosis, brain region, age, and sex. Overall, DCRNet ranks first in 27.08% of all biological conditions, compared to 31.25% for Granger, 29.17% for seqICPnl, and 12.50% for Cycle (Fig. 8b). To assess the statistical significance of these performance differences, pairwise Wilcoxon signed-rank tests are performed, with *P*-values adjusted using the Benjamini–Hochberg correction. No statistically significant differences are observed among the three sequential causality methods after multiple testing correction (DCRNet versus Granger: adjusted *P* = .3092, DCRNet versus seqICPnl: adjusted *P* = .0675, Granger versus seqICPnl: adjusted *P* = .0566). However, these three methods significantly outperform the correlation-based method Cycle (DCRNet versus Cycle: adjusted *P* = 4.09E-08, Granger versus Cycle: adjusted *P* = 4.09E-08, seqICPnl versus Cycle: adjusted *P* = 7.75E-04). Overall, the performance of DCRNet across diverse biological contexts supports its utility in generating lncRNA regulatory hypotheses.

**Figure 8:**
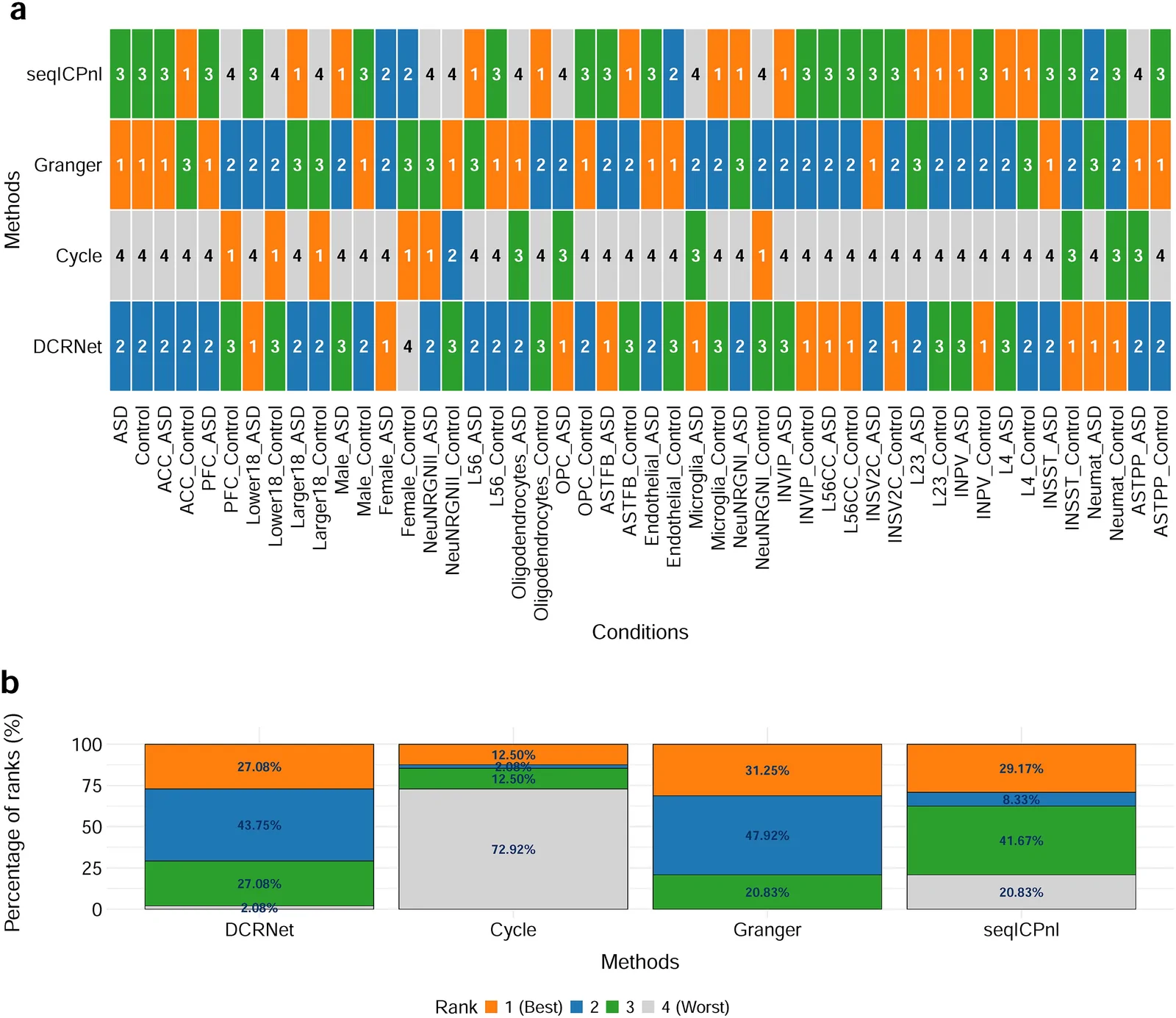
Comparison of DCRNet with Cycle, Granger causality, and seqICPnl in inferring lncRNA-mRNA causal relationships across 48 biological conditions. (a) Performance ranking of each method. (b) Distribution of ranks across all conditions.

By performing differential causal network analysis between ASD and control, we have identified one ASD susceptibility lncRNA (*LINC02712*) for Cycle, 11 (*BCYRN1, MAST3-AS1, ABHD15-AS1, MFSD12-AS1, TRMT2B-AS1, LINC00903, INPP5B-AS1, EPC1-AS1, LINC00685, SNHG9, SNHG31*) for Granger and 6 (*BCYRN1, ABHD15-AS1, MAST3-AS1, LINC00685, TRMT2B-AS1, LINC01632*) for seqICPnl, all exhibiting statistically significant susceptibility scores (Supplementary Table S20). Comparing these ASD susceptibility lncRNAs with cell type-specific differential expression results from an independent snRNA-seq dataset that profiles the same cortical cell types [59, 60], we find that the only lncRNA identified by Cycle is not validated, whereas 4 of the 11 lncRNAs (*ABHD15-AS1, LINC00903, LINC00685, SNHG9*) for Granger and 3 of the 6 lncRNAs (*ABHD15-AS1, LINC00685, LINC01632*) for seqICPnl are independently validated as differentially expressed in at least 1 cell type in ASD versus control. Overall, compared with Cycle, Granger, and seqICPnl, DCRNet identifies eight ASD susceptibility lncRNAs, of which four are independently validated, yielding a validation rate of 50%. This suggests that DCRNet achieves a validation rate comparable to seqICPnl and exceeds those of and Granger and Cycle, supporting its utility in predicting ASD susceptibility lncRNAs.

We next assess the overlap between the rewired lncRNA-mRNA pairs and the DEGs from the same ASD cortical dataset [28]. In the ASD-associated context, DCRNet covers 270 of 513 cell type-specific DEGs (52.63%), marginally higher than Granger (264 of 513, 51.46%), followed by seqICPnl (86 of 513, 16.76%) and Cycle (2 of 513, 0.39%). In the region-dependent context, DCRNet and Granger show comparable coverage of ACC-specific DEGs, with DCRNet covering 84 of 130 (64.62%), Granger covering 81 of 130 (62.31%), seqICPnl covering 19 of 130 (14.62%), and Cycle covering 0 of 130 (0.00%). For PFC-specific DEGs, DCRNet identifies 38 of 67 (56.72%), Granger identifies 40 of 67 (59.70%), seqICPnl identifies 9 of 67 (13.43%), and Cycle identifies 0 of 67 (0.00%). These results indicate that DCRNet effectively identifies rewired lncRNA-mRNA pairs that converge on DEGs, particularly in the ASD-associated context, while maintaining competitive performance in the region-dependent context. The complete sets of overlapping pairs are provided in Supplementary Tables S21 and S22.

## Discussion

In this work, we present DCRNet, a hidden causality-based framework designed to infer dynamic lncRNA causal regulation in ASD. While DCRNet builds upon established methods (metacell construction, trajectory inference, pattern causality, and differential network analysis), its value lies in the biological discoveries enabled by their systematic integration. Our analyses show that eight ASD susceptibility lncRNAs are significantly involved in ASD pathogenesis. Furthermore, lncRNA expression signatures distinguish sex and cell types, suggesting their potential as candidate biomarkers. We identify 20 immune-related lncRNAs with potential links to neuroimmune regulation, along with differences in cell–cell interaction networks between ASD and control cohorts. DCRNet shows performance comparable to Granger and seqICPnl in capturing dynamic lncRNA regulation and outperforms the correlation-based approach Cycle. Despite these findings, several limitations inherent to DCRNet should be considered.

The concept of dark causality, which captures mixed positive and negative regulatory effects of an lncRNA on the same target, is supported by known biological precedents, including the *METEOR* and *HAUNT* genetic loci [26, 27], which exhibit developmental stage-dependent regulatory switches. These examples illustrate context-dependent directional switching rather than simultaneous opposing regulation, which is precisely the type of dynamic behavior that dark causality aims to capture from pseudo-temporal data. In our analysis, dark causality is a prevalent regulatory mode across diverse biological contexts. Notably, genes regulated through dark causality are significantly enriched for neurodevelopmental and ASD-related pathways, suggesting that this pattern operates on functionally relevant processes rather than arising as a mere artifact of the inference framework. Nevertheless, this concept remains in its early stages of characterization and should currently be interpreted as a robust statistical observation across our dataset rather than a fully validated biological mechanism. Future work, including time-series perturbation assays and single-cell tracking of lncRNA-target dynamics, will be valuable for testing its biological validity.

In SuperCell [30], the construction of metacells involves three critical parameters (the number of nearest neighbors, the number of the top genes, and the graining level) that influence the balance between biological resolution and computational robustness. To prioritize fine-grained local structure, the number of nearest neighbors to build KNN (K-Nearest Neighbors) network is set to 5. The use of the top 2000 variable genes for dimensionality reduction is consistent with common practices in snRNA-seq data and supports efficient computation without substantial loss of biologically relevant information. A graining level of 30 is to define the approximate number of cells aggregated per metacell, and it achieves a practical trade-off between mitigating technical noise and preserving biological relevance. To ensure reliable downstream analysis (trajectory inference and hidden causal inference), future efforts will focus on enhancing the rigor of metacell partitioning in snRNA-seq data analysis.

For the application of trajectory inference to metacells, DCRNet applies a linear trajectory inference method SCORPIUS [32], which assumes simplified, unidirectional transitions between cell states. This choice is driven by the downstream causal inference, which requires a single, unambiguous temporal coordinate per cell. Unlike nonlinear trajectory inference methods that explicitly model branching events, such as Monocle3 [75], Slingshot [76], and PAGA [77], SCORPIUS explicitly avoids assigning multiple pseudotime values to cells near bifurcations, which would violate the identifiability assumptions of our causal models and introduce irreducible ambiguity. While SCORPIUS is interpretable and efficient, it fails to capture complex biological processes, such as branching events, cyclic dynamics, or convergent differentiation pathways in ASD. Moreover, beyond the choice of a specific trajectory inference method, causal inference based on pseudotime reconstruction more generally remains a subject of ongoing debate and is sensitive to preprocessing choices. The pseudotime-based ordering should, therefore, be viewed as a computational approximation of relative cell states rather than as a definitive temporal sequence, and that the resulting regulatory inferences require experimental validation. In future work, DCRNet will adopt nonlinear approaches to order metacells and quantify the resulting topological distortion by comparing our linear ordering with the dominant branch. Experimental designs that enable genuine temporal sampling, such as time-series perturbation assays, would also be valuable to move beyond pseudotime-based inference.

The parameter settings of hidden causal inference in DCRNet are carefully determined to balance computational feasibility and biological interpretability. The embedding dimension *E* = 3 and time lag *τ =1* are selected in accordance with Takens’ theorem for state-space reconstruction [78]. We select Euclidean distance metric due to its widespread applicability and robustness in capturing distance within reconstructed state spaces. The number of steps ahead *h* = 1 aims to infer immediate causal relationships, which is particularly meaningful given rapid responses in gene regulatory networks. A critical empirical parameter is the causal strength cutoff set at 0.60, used to identify significant lncRNA-mRNA causal relationships. This threshold seeks to minimize false positives while retaining biologically plausible causality. Future studies could benefit from applying adaptive cutoff strategies.

While data-driven hidden causal inference framework DCRNet can identify temporal causality, it is often susceptible to false positives due to high-dimensionality and noise in snRNA-seq data. To enhance the accuracy and biological interpretability of inferred lncRNA causal regulatory networks, we incorporate the priori information of lncRNA targets predicted by LncTar [33]. LncTar relies solely on thermodynamic free energy minimization, which is biologically restrictive because many lncRNAs function through RNA-binding proteins or chromatin-modifying complexes rather than direct base-pairing. Nevertheless, we chose LncTar for its demonstrated reliability (80% validation against experimentally supported interactions [33]) and scalability for systematic screening. Importantly, these predictions serve as a priori filter to guide causal inference rather than as the sole basis for our findings. Future refinements could stratify these predicted targets into confidence levels by integrating orthogonal evidence from other databases (e.g. RegNetwork [58], NPInter [79], and starBase [80]).

By providing single-nucleus resolution, snRNA-seq captures the full spectrum of cellular diversity in the brain through detailed transcriptional profiles. However, it may suffer from lower transcript capture efficiency and higher technical noise, particularly for lowly expressed lncRNAs. In contrast, bulk RNA sequencing offers high detection sensitivity for transcripts, especially low-abundance lncRNAs. In future, integrating snRNA-seq and bulk RNA sequencing data provides a powerful strategy to enhance the inference and functional interpretation of lncRNA causal regulation in ASD.

The comprehensive evaluation of heterogeneity between networks is essential for quantifying structural and functional divergence in systems biology. In terms of lncRNA causal regulatory networks, we use Simpson index [81] to evaluate the heterogeneity between different brain biological contexts. Actually, the Jaccard index [82] and Lin’s method [83] can also be used to characterize network heterogeneity. Applying a multi-metric index (combining Simpson index, Jaccard index, and Lin’s method) would enhance interpretability and robustness in characterizing network heterogeneity, especially in ASD.

Our classification analysis is designed to evaluate the discriminative power of lncRNA biomarkers within DCRNet. While lncRNA biomarkers distinguish sex and cell types with high accuracy in our dataset, the classification performance is moderate for diagnosis, brain region, and age. For example, the lncRNA biomarker set for distinguishing ASD from control achieves an accuracy of 0.5735, an AUC of 0.6065, and an F1 score of 0.5477. For classifying brain region, the classification model has an accuracy of 0.6589 (AUC: 0.6524, F1: 0.7755) within ASD and 0.6538 (AUC: 0.7147, F1: 0.7433) within control. Age prediction yields accuracies of 0.7020 (AUC: 0.6080, F1: 0.8210) in ASD and 0.6945 (AUC: 0.6765, F1: 0.8021) in control. This indicates that although potential lncRNA biomarkers can clearly distinguish basic biological categories (e.g. sex and cell type), their ability to differentiate systems-level traits (e.g. diagnosis, brain region, and age) is still limited. Such a discrepancy is consistent with the known biology in that sex-biased brain gene expression represents a robust, evolutionarily conserved program [84], and cell-type identity is encoded by well-defined transcriptional programs that dominate single-cell variance [85]. In contrast, ASD-associated transcriptomic alterations are typically of moderate-to-small effect size and highly variable across individuals [86], cortical transcriptomic differences are predominantly graded and cell-type-proportional rather than discrete [87], and age-dependent transcriptional changes follow a continuous trajectory that a binary split artificially fragments [88], all of which collectively limit the discriminative power of these systems-level traits. From a computational perspective, evaluating additional classification algorithms would be a promising direction for future work to further optimize predictive performance for these systems-level traits.

Although the lncRNA biomarker set for diagnosis yields moderate performance in distinguishing ASD from control cohorts, nearly half of the lncRNAs (26 of 60 context-specific and 22 of 42 context-enhanced biomarkers) comprising the diagnostic signature are independently identified as differentially expressed in at least one cortical cell type in an external snRNA-seq dataset [59, 60] (Supplementary Table S23). This convergent evidence suggests that, while insufficient as a standalone classifier, these lncRNAs may capture signals that are consistent with ASD-associated molecular changes and are reproducible across the cohorts examined. Of note, *PPP1R26-AS1, TMEM72-AS1, FAM66C, ZEB1-AS1, NEAT1, MALAT1*, and *SNHG6*, seven of the lncRNA biomarkers identified across our classification analyses (diagnosis, region, age, sex, and cell type), have also been reported among blood-derived or differentially expressed lncRNAs (adjusted *P* < .05, adjusted by the Benjamini–Hochberg method) [14, 20, 62, 89], providing additional cross-tissue support for their biological relevance.

The predominance of antisense lncRNAs among the identified ASD susceptibility and immune-related sets likely reflects several factors. Genomically, ~40% of ASD risk loci express natural antisense transcripts, indicating a structural enrichment of antisense-mediated regulation [90]. Functionally, antisense lncRNAs achieve high regulatory specificity through direct base-pairing with sense targets, and several validated ASD-associated antisense lncRNAs, such as *MSNP1AS* and *PTCHD1-AS*, illustrate this regulatory mode [91, 92]. This intrinsic specificity, together with the known enrichment of antisense transcription in the mammalian brain, likely explains why antisense lncRNAs dominates lncRNA regulation in ASD.

We benchmark DCRNet against Granger and seqICPnl as representative causal inference methods, and against Cycle as a correlation-based baseline. In terms of validation, we note that our approach relies on RegNetwork [58] as a reference database, which is inherently incomplete and may not capture all true lncRNA-mRNA regulatory relationships. Furthermore, the percentage of recovered interactions alone does not fully capture predictive accuracy. While the DEG overlap analysis from the same transcriptomic cohort provides biological context for the inferred rewired pairs, we have also performed orthogonal validation using independent datasets and literature. Nevertheless, we recognize that more comprehensive benchmarking, including synthetic data simulations and additional performance metrics such as precision-recall analysis, would further strengthen the evaluation of DCRNet. More broadly, benchmarking against a wider range of state-of-the-art tools will be a useful direction for future work.

DCRNet holds significant potential for extension to other regulatory ncRNAs, including microRNAs (miRNAs), circular RNAs (circRNAs), and Piwi-interacting RNAs (piRNAs). By refining priori information of ncRNA targets and tuning parameter settings to match ncRNA-specific causal regulatory dynamics, DCRNet could evolve into a unified causal inference platform for diverse regulatory ncRNAs. This could facilitate the exploration of multilayer ncRNA regulatory networks in ASD and support the discovery of candidate ncRNA biomarkers or therapeutic targets.

Taken altogether, we develop a computational framework DCRNet to delineate dynamic lncRNA causal regulation in ASD. Our findings suggest the context-specific heterogeneity of lncRNA causal interactions across the biological contexts examined, offering insights into their regulatory roles in ASD. We anticipate that DCRNet will be applicable to exploring lncRNA regulatory mechanisms in other neurodevelopmental contexts. Nevertheless, all regulatory predictions presented in this study are computational and require experimental validation. The identified ASD susceptibility lncRNAs, biomarker candidates, and altered cell–cell communication networks should therefore be interpreted as hypothesis-generating resources to guide future mechanistic studies.

**Key Points**

- DCRNet is developed to delineate dynamic lncRNA regulation in ASD.
- Eight ASD susceptibility lncRNAs may be linked to disrupted gene regulatory programs.
- Long non-coding RNA expression signatures suggest potential for classifying sex and cell type.
- Twenty immune-related lncRNAs are predicted to be involved in neuroimmune regulation.
- Cell–cell interaction networks show notable differences between ASD and control cohorts.

## Supplementary Material

Supplementary_material_bbag541

## Data Availability

The source code for DCRNet is available at https://github.com/zhangjunpeng411/DCRNet. All accession codes, unique identifiers, and web links for publicly available datasets are described in the paper. All data supporting the findings of the current study are listed in Supplementary material.
